# ‘Analogy-Based Comprehensive Diabetes Education’ (ABCDE) Improves Glycemic Control of Diabetic Patients in an Underserved Population: Results of a Retrospective Chart Analysis

**DOI:** 10.3390/healthcare10030409

**Published:** 2022-02-22

**Authors:** Rajagopal V. Sekhar

**Affiliations:** Section of Endocrinology, Diabetes and Metabolism, Baylor College of Medicine, Houston, TX 77030, USA; rsekhar@bcm.edu

**Keywords:** diabetes, analogies, education, glycemic outcomes

## Abstract

Diabetes is the leading global cause for blindness, kidney failure and amputations. Preventing these complications requires optimal glycemic control, and it is imperative that diabetic patients understand the fundamental concepts of diabetes care. Although patients attend formal diabetes education classes, many do not comprehend basic concepts of diabetes, and are often noncompliant with diet, exercise and medications. A novel approach termed ‘analogy-based comprehensive diabetes education’ (ABCDE) was developed to educate HIV-patients with diabetes about basic concepts of diabetes care. The object of this manuscript is to report the results of a retrospective chart review on the impact of ABCDE on glycemic outcomes in 24 patients who had failed usual care (including formal diabetes education, physician visits, and diabetic medications), and were non-adherent with diet and medications. They received only the ABCDE without any changes in pharmacotherapy. The impact on glycosylated hemoglobin (HbA1c) and fasting blood glucose (FBG) was assessed at subsequent visits. HbA1c was found to decline by 22% and 33% after 3 and 6 months, respectively, with corresponding declines in FBG by 53% and 59%, respectively. These results suggest that ABCDE in outpatient diabetes clinics could be effective in behavior modification toward improving glycemic control, and warrants additional investigation.

## 1. Introduction

According to the World Health Organization (WHO), diabetes was responsible for a 5% increase in premature mortality between 2000–2016, and was the 9th leading cause of death in 2019 [1]. The prevalence of diabetes is growing and predicted to affect 522 million people worldwide by 2030, with the healthcare expenses for managing diabetes and its related complications exceeding 825 billion dollars annually by 2030 [2].

Diabetes care usually involves a multidisciplinary team which provides diabetes education, nutrition advice, pharmacotherapeutic management (provided by nurse practitioners, physician-assistants, primary care physicians and endocrinologists), and annual retinal scans by ophthalmologists. A key starting point for providing diabetes care formal diabetes education which is usually provided by certified diabetes educators in classroom style sessions, either by individual classes or group classes. Components of diabetes education address basic information about hyperglycemia, definitions of diabetes, components of diabetes management including lifestyle interventions and pharmacotherapy, knowledge about assessment of glycemic status via glycosylated hemoglobin and other relevant lab work, knowledge about home glucose monitoring, diabetes complications and the importance of good compliance and regular follow-up [3]. Such formal diabetes education is intended to improve awareness and provide understanding toward healthcare practices and behavioral improvement in patients with diabetes [4], with the ultimate goal of improving adherence with overall diabetes self-management by patients. The WHO defines ‘adherence’ as ‘the extent to which a person’s behavior–taking medication, following a diet, and/or executing lifestyle changes, corresponds with agreed recommendations from a health care provider’ [5]. However, poor adherence with following a healthy diet, exercising and taking medications remains a significant barrier to achieving optimal glycemic control in patients with diabetes [6,7,8,9,10], with non-attainment of glycemic goals [11] resulting in diabetic complications [12], increased hospitalization and mortality [13]. 

Although improved patient adherence improves glycemic control [14], substantial barriers still exist [15]. Health disparities in different racial groups with regards to diabetes result in worse outcomes and higher mortality rates despite similar incidence of these disorders [16]. Barriers to achieving optimal glycemic control include socioeconomic factors [17,18], linguistic barriers [19,20], and race/ethnic barriers [21,22,23,24]. These barriers contribute to higher complications and mortality in diabetic patients [12,13], and efforts are being continually made to understand and eliminate racial and ethnic disparities in healthcare [25]. Many of these barriers occur in minorities with diabetes [26] and especially in patients with HIV, which typically combines racial/ethnic barriers, socioeconomic barriers and linguistic barriers [27]. Although formal diabetes education is reported to be impactful on the practice and behavior of diabetic patients [28], this is not evenly observed in all diabetic populations. In underserved populations such as HIV patients with economic, racial, ethnic and linguistic barriers, diabetic patients often tend to get overwhelmed, and struggle to fully understand information critical to their success in achieving optimal control and the result ranges from indifference to anxiety, frustration, mistrust and rejection of expectations, which result in poor adherence to lifestyle measures and medications [6,7,8,9,10]. 

Could an alternate simpler approach of providing the same diabetes education improve adherence and glycemic control? An important component of the self-management of any chronic disease is health literacy. The Centers for Disease (CDC) defines health literacy as ‘the degree to which individuals have the ability to find, understand, and use information and services to inform health-related decisions and actions for themselves and others’ [29]. This definition incorporates the ability to use health information, make well-informed decisions, and acknowledges that organizations have a responsibility to address health literacy and incorporate a public health perspective [29]. Poor health literacy has been suggested as a reason to explain poor outcomes and racial disparities in diabetes medication adherence [30,31]. For diabetic patients, health literacy involves the need to first understand the components of diabetes to improve self-care and make effective healthcare decisions. More and effective physician communication with diabetic patients was proposed as the solution [32], with the need for ‘thinking outside the box’ approaches to improve patient education about diabetes [33,34], and a recent publication proposes that tailoring educational approaches based on patients’ psychological phenotypes may be needed to promote optimal self-management behaviors [35]. Consistent with these publications encouraging a rethink on how to provide diabetes education and improve self-management, an alternate, experimental ‘analogy-based’ approach has been developed and is in clinical use to provide diabetes education to an underserved population of HIV patients with diabetes, toward providing diabetes education in a simple and effective manner. This is known as the ABCDE (‘analogy-based comprehensive diabetes education’) approach. Patients referred to the diabetes clinic had already received formal diabetes education by a certified diabetes educator, and remained non-adherent with diabetes self-management necessitating referral by the primary care physician to the diabetes clinic where they received the ABCDE. This specific objective of this retrospective chart analysis was to test the hypothesis that HIV patients with diabetes who received the ABCDE approach alone would improve clinical glycemic parameters (glycosylated hemoglobin, estimated average glucose and fasting blood glucose levels) 3 months and 6 months after receiving the ABCDE. 

## 2. Materials and Methods

### 2.1. Ethics

This study is a retrospective chart analysis performed after obtaining approval by the Institutional Review Board. 

### 2.2. Study Protocol 

Data from retrospective analysis of charts from patients with type 2 diabetes attending a specialist diabetes clinic serving HIV-infected patients from low socioeconomic strata are reported. Such patients referred to the specialist diabetes clinic already have ‘usual care’ which includes received formal diabetes education and diabetic pharmacotherapy from the primary care physicians. Such patients were referred because they failed ‘usual care’ and had uncontrolled hyperglycemia. At their initial visit, several of these patients reported that they although they attended formal diabetes education classes and have heard of terms such as ‘HbA1c’, they did not comprehend how it affects their health, and only recognize it as something ‘bad’. They were unfamiliar with basic concepts and needs for self-diabetes management, and were poorly compliant with diet, exercise and especially medication. Such patients reported being helpless and frustrated in not being able to improve their health and being repeatedly asked to improve by their healthcare providers. To overcome these barriers in understanding without the need to replace diabetes information to be communicated (i.e., to change the language, not the message) this author developed a novel ‘analogy’-based education where basic key concepts of diabetes would be compared to common everyday events in a patient’s life which they could relate to and apply toward the understanding of diabetes. This ‘analogy-based comprehensive diabetes education’ was termed the ABCDE approach, and used as a clinical teaching approach. It was enthusiastically received by diabetic patients who expressed a much better understanding of basic concepts of diabetes and self-care for the first time. Based on this experience, this manuscript outlines the ABCDE approach, and the impact that receiving ABCDE had on glycemic outcomes in 24 patients with hyperglycemia in whom no other changes were made. Because these patients already had active prescriptions and medications but were not adherent with taking them, no changes in medications or increases in doses were made—instead, patients were provided the ABCDE and asked to take their prescribed medications, improve diet, and diabetes-related self-care.

After obtaining approval by the Institutional Review Board (IRB), a retrospective chart analysis was performed to collect glycemic data from 24 patients (12 men, 12 women) who visited a specialist HIV-diabetes clinic serving an underserved population of HIV patients from 2014–2018 (before the COVID pandemic). The pre-pandemic timeframe was chosen to keep the healthcare background uniform (because of the multiple variations of how healthcare was disrupted and altered with initiation of telephonic and other forms of visitations). 

Inclusion criteria: (1) Presence of type 2 diabetes; (2) report of poor adherence with diabetic medications (defined as missing doses at least twice per week) and diet; (3) access to diabetic medication. 

Exclusion criteria: (1) Any patient receiving ABCDE for whom medication was changed (defined here as dose changes, or new medication added); (2) lack of receiving prior formal diabetes education or visit to dietitian; (3) use of nondiabetic medications which could aggravate glycemia (example, corticosteroids); (4) brain pathologies such as stroke or dementia; (5) lack of access to diabetic medication. Such patients were provided ABCDE and asked to take their medication as prescribed and follow a healthy diet (as previously instructed at a dietitian visit). The retrospective analysis captured glycemic data from their electronic medical records and included data about the glycosylated hemoglobin (HbA1c) test and fasting blood glucose values at baseline (at the time of the first visit) and the subsequent two visits, typically at 3 months and 6 months after the first visitation. In this report, Visit-1 refers to the initial visit, and Visit-2 and Visit-3 refer to subsequent visits 3- and 6 months after Visit-1. 

### 2.3. Details of ABCDE

ABCDE is an acronym for ‘analogy-based comprehensive diabetes education’. Basic components of diabetes education involve understanding the importance of compliance with diet, exercise, taking medications and having an annual eye exam. Furthermore, it is critically important that patients understand what the HbA1c test is, so that they can understand the true implications of uncontrolled hyperglycemia and improve self-care. 

#### 2.3.1. The ‘Chair Analogy’ for Following Diet, and Taking Exercise and Medication

Well-controlled diabetes is compared to a chair supported by 4 strong legs. The patient is asked what would happen if, when they are sitting on such a chair, any one leg of the chair would break, and their reply is the they would fall. The point is emphasized by the physician that it does not matter which leg of the chair were to break, the result will be that the chair would topple. Then, the patient is asked what would happen if 2 or more legs break, and the answer again is that they would fall. Then, the analogy is connected to diabetes, when the patient is told that the chair represents well-controlled diabetes, and each leg of the ‘diabetes chair’ represents one aspect of diabetes management. One leg represents the liquids they drink, a second leg represents food, a third leg represents exercise and the fourth leg represents medications. Similar to how a stable chair requires 4 sturdy legs, similarly all 4 components of diabetes care need to be followed for optimal glycemic control.

#### 2.3.2. The ‘Vehicle-Fuel Analogy’ for Avoiding Sugary Beverages

Since many diabetic patients consume sugary beverages, fruit juices and sugar calories in bottled or canned drinks, there is a basic lack of understanding/interest in the importance of avoiding these drinks and to drink water. Most patients in the clinic drive to the clinic, and are familiar with a car/motor vehicle. They are asked what fuel they use for a vehicle they currently own, or have owned in the past, or are familiar with, and the reply is mostly that gasoline is used. Then, they are asked if they have ever filled a non-gasoline fuel such as diesel by mistake, and the usual response is that they have never made such a mistake. Then they are asked what would happen if they hypothetically filled diesel in a car that runs on gasoline (or the converse), and the answer circles around the fact that it would damage the engine, and the car will not run. Then, the analogy is made that just like their car that can only ‘drink’ and run on gasoline (and not diesel), our human bodies are designed to run on water, and not sodas/sugary drinks/fruit juices. Just like filling diesel in a gasoline car will create problems, drinking sugary beverages will cause problems. Some patients ask if it is appropriate to drink their favorite sugary beverages intermittently rather than giving them up, and their question is answered with a question asking if it is appropriate to fill diesel in their gasoline car intermittently. Their answer is resolute in saying ‘never’, and they make the connection that the answer to their question is also ‘never’.

#### 2.3.3. The ‘Bucket Analogy’ for Improving Compliance with Diet

Many patients are under the mistaken impression that as long as they take their medication, that is all they need to do. Such patients do not place importance on the value of a healthy diet, and often make mistakes including habitual consumption of fast food, sweets and unhealthy food, and are frustrated when glycemic goals are not achieved despite taking medications. The ‘bucket analogy’ is an educational attempt to help patients realize that it is not enough to only be compliant with medications, but it is equally important to follow a healthy diet. The patient is asked if they are given an empty bucket, how would they fill it? The typical answer is that they will fill it using a water faucet. They are then asked that if another empty bucket is given to them with 3 large holes at the bottom of the bucket, how would they fill such a bucket with water. They answer is that it is not possible to fill such a bucket as all the water would leak out of the 3 large holes at the bottom of the bucket. Then, they are asked whether using more than 1 faucet can make a difference, and the answer remains that it is not possible to fill this bucket with holes no matter how many faucets are used. Then, the analogy is made to diet—the filling of the bucket represents glycemic control, each faucet represents one medication, and that each hole in the bucket represents a dietary mistake they are doing (eating fast food, sweets etc.). Just as it is difficult to fill a bucket with water when it has holes (no matter how many faucets are used), it is difficult to control diabetes when they do not follow a good diet, no matter how many medications are used. 

#### 2.3.4. The ‘Speedometer Analogy’ to Explain Glycosylated Hemoglobin

Many patients have heard of the HbA1c blood test, but do not grasp the vital importance of how higher HbA1c numbers are detrimental to their health. This analogy was developed to help patients understand what the HbA1c test represents, and resonates well with patients who are familiar with the legal requirement of speed limits for vehicles in school zones. Patients are asked what the typical speed limit in a school zone is, and the answer is usually 20 miles per hour. They are asked why it is important to drive so slowly in a school zone during active hours, and they respond appropriately, that it is for safety reasons because there are many children and parents around, and it is possible to stop quickly if someone inadvertently comes in front of their vehicle. Then, they are asked how they know they are driving at the required speed of 20 miles per hour, and the response is that they look at the speedometer to be sure they are driving at the legally required speed limit. Then, there is a switch to an analogy where patients are asked to pretend that the disease of diabetes represents a car, and the HbA1c test is like the speedometer of the ‘pretend’ diabetes car. Just as in the real world the correct speed limit in an active school zone is 20 miles/h, the speed limit of the ‘pretend diabetes car’ is a HbA1c test value of 7%. They are asked to imagine that each 1% of HbA1c value represents an additional 20 miles/hour of driving speed, and are asked to count along with the physician. They count as follows: 7% is 20 mph, 8% is 40 mph, 9% is 60 mph, 10% is 80 mph, 11% is 100 mph and so on. Then their HbA1c test result is revisited. If, for example the patient realizes that their HbA1c test was 11% they understand that it is similar to driving their car at 100 miles/h in an active school zone full of children. They realize the real potential for disaster in this imaginary example, and this realization powerfully translates to how poorly controlled their HbA1c is and what it could represent in terms of health. Many patients report that this analogy helped them understand for the first time in their lives how serious this situation is.

#### 2.3.5. The ‘Close-Your-Eyes Analogy’ for the Importance of Annual Retinal Scans

Often times patients do not recognize the necessity of getting an annual retinal scan. Although patients have heard that poorly controlled diabetes carries an increased risk of blindness, this remains an abstract concept. The ‘close-your-eyes analogy’ is a simple way of communicating the importance of getting an annual retinal scan. Seated patients are asked to close their eyes, and asked what they see. They typically answer that they cannot see anything, and are informed that this is what complete blindness could represent. It is to avoid this serious complication, that annual retinal scans are needed so that any emerging retinal damage can be addressed at an early stage. 

### 2.4. Statistical Analysis

Results are reported as means ± SD (standard deviation). A repeated measures analysis of variance (ANOVA) with Bonferroni multiple comparisons test was used for the statistical analyses to compute differences in means between results at visit 2 and visit 1, and visit 3 and visit 1 for glycosylated hemoglobin (HbA1c), estimated average glucose and fasting blood glucose concentrations. Results are statistically significant at *p* < 0.05. 

## 3. Results

### 3.1. Patient Information

All patients in this retrospective report attended the Diabetes Clinic at the HIV health center, a community outpatient healthcare provider for underserved patients with HIV infection. There were 12 women and 12 men, and none had a college degree. All participants had established care with a HIV primary care provider, and had received ‘usual care’ comprising of medical outpatient clinic visits, diabetes education and nutritional counseling, prior to being referred to the Diabetes Clinic for poor diabetes control. 

### 3.2. Changes in Glycemic Indices to the ABCDE

**Figure 1:**
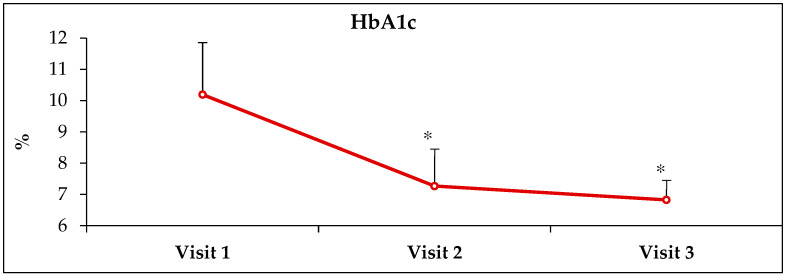
**Improvement in glycosylated hemoglobin (HbA1c) after receiving ABCDE.** Visit-1 = first visit where ABCDE was provided; Visit-2 and Visit-3 = visits 3- and 6 months after Visit-1. * = *p* < 0.00001.

**Figure 2:**
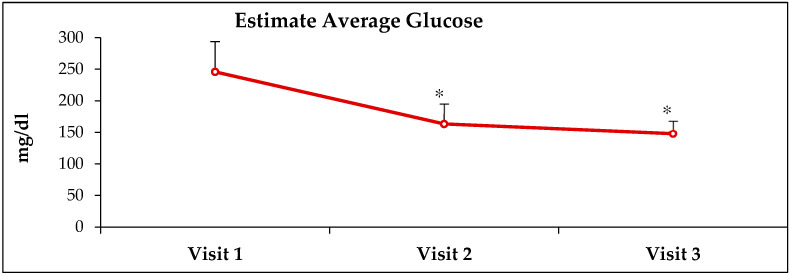
**Improvement in estimated average glucose after receiving ABCDE.** Visit-1 = first visit where ABCDE was provided; Visit-2 and Visit-3 = visits 3- and 6 months after Visit 1. * = *p* < 0.00001.

**Figure 3:**
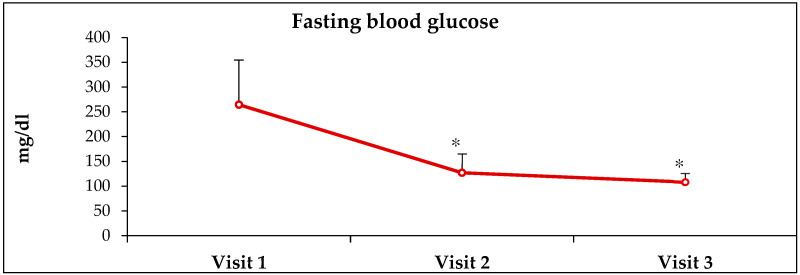
**Improvement in glycemic indices after receiving ABCDE.** Visit-1 = first visit where ABCDE was provided; Visit-2 and Visit-3 = visits 3- and 6 months after Visit 1. * = *p* < 0.00001.

**Figure 4:**
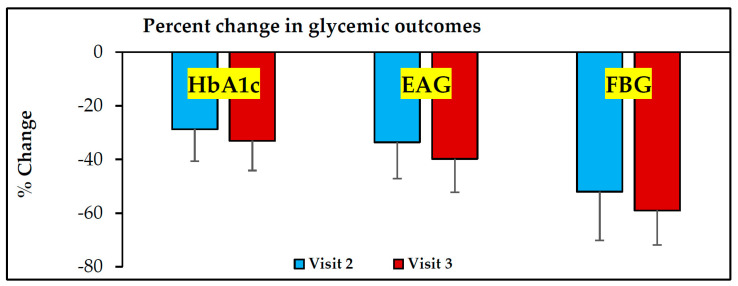
**Percent decline in glycemic indices after receiving ABCDE.** Visit–1 = first visit where ABCDE was provided; Visit-2 and Visit-3 = visits 3- and 6 months after Visit–1. HbA1c = glycosylated hemoglobin, EAG = estimated average glucose, FBG = fasting blood glucose.

HbA1c, estimated average glucose (EAG) and fasting glucose (FBG) values were obtained from electronic medical records from Visit-1 (before receiving ABCDE, Visit-2 (3 months after receiving ABCDE) and Visit-3 (6 months after receiving ABCDE) (Table 1). At Visit-2, there was a decrease of 28.7% (*p* < 0.0001), 33.6% (*p* < 0.0001) and 52% (*p* < 0.00001 in HbA1c, EAG and FBG values, respectively, when compared to pre-ABCDE values at Visit-1 (Figure 1, Figure 2, Figure 3 and Figure 4). At Visit-3, these outcomes decreased further with 33.1% (*p* < 0.0001), 39.8% (*p* < 0.0001) and 59.0% (*p* < 0.0001) in HbA1c, EAG and FBG compared to pre-ABCDE values at Visit-1 (Figure 1, Figure 2, Figure 3 and Figure 4). However, differences between Visit-2 and Visit-3 were more modest with improvements of 6.1% (*p* = 0.11), 9.3% (*p* = 0.06) and 14.6% (*p* = 0.07) in HbA1c, EAG and FBG, respectively. Because these improvements occurred without a change in medication (i.e., patients were asked to continue medication and follow the lifestyle advice that they had already received), the improvements at Visit-2 indicate behavioral improvement, and the continued improvement at Visit-3 suggests that the impact of ABCDE on improving patient compliance with lifestyle measures and medication compliance lasted over a longer duration of time.

## 4. Discussion

This manuscript reports glycemic indices on a retrospective chart analysis from HIV-patients of a lower socioeconomic background attending a specialist diabetes clinic. Patients who reported limited adherence with lifestyle measures and medications despite having received usual care from primary physicians and diabetes educators were provided the ‘analogy-based comprehensive medical education’ (ABCDE). They were asked to improve self-care, and medication adherence. Because these patients had access to diabetic medications but were poorly adherent with taking them as prescribed, no increases in medication doses were advised and no new medications were added. Instead, patients were asked to take their prescribed diabetes medications as advised and follow lifestyle measures of diabetes self-care. The key findings of this retrospective chart analysis are that after receiving ABCDE, glycemic levels improved dramatically 3- and 6 months after receiving ABCDE. 

### 4.1. Lack of Attainment of Glycemic Goal in Patients with Diabetes

Despite the availability of diabetes care from physicians, physician-assistants, nurse practitioners, certified diabetes educators and nutritionists, a majority of patients are reported to not reach targets indicative of excellent glycemic control [11,14], mostly due to non-adherence with the components of self-care including lifestyle measures and pharmacotherapy [6,7,8,9,10]. This results in higher rates of diabetic complications, hospitalizations and mortality [12,13]. Multiple barriers are involved in diabetic ‘non-adherence’ [15] including health disparities [16], socioeconomic factors [17,18], language [19,20], race and ethnicity [21,22,23,24,25], and these barriers especially impact minority and underserved populations, such as HIV patients [26,27]. Improving patient understanding and facilitating behavior modifications are important [28], but have been a challenge and difficult to achieve.

### 4.2. Pitfalls with Formal Diabetes Education

According to published reports, many diabetic patients do not meet established glycemic goals [11,14]. Diabetic patients from a lower socioeconomic status who are referred to specialist diabetes outpatient clinics for ‘brittle diabetes’ or ‘complicated diabetes’ have already received formal diabetes education, but often have not understood basic terminology or concepts, and this is especially relevant when there are additional linguistic barriers [19,20]. This prevents patients developing ‘health literacy’ about basic concepts of diabetes [29,30,31], i.e., they have attended classes to receive diabetes education but have understood it poorly and thus, lack the ability to use the information to improve self-care and make good healthcare decisions. In the patients reported in this paper, it was evident during their first visit to the diabetic clinic that they had not understood what the glycosylated hemoglobin test was, or why it was important to stop drinking sugary beverages, eat healthy, exercise, take medications regularly without missing doses, or get annual retinal exams. This lack of connection that patients had regarding their diabetes, despite having attended formal diabetes education classes, was seen as a significant barrier to achieving optimal glycemic control. 

### 4.3. Why ABCDE Was Developed and Why It Works

Targeted education provided by multiple formats has been shown to improve outcomes in patients with diabetes [32,33]. When the current modalities of providing diabetes education is examined, it is clear that there is scope for confusion in understanding complicated medical terminology. An important example to illustrate this point comes from how educators and providers communicate what the ‘glycosylated hemoglobin’ is, this is usually explained as an averaged blood glucose value over a 3-month timeframe. Although this explanation is perfectly clear to healthcare providers as a factually realistic explanation, it is an abstract concept to those without a healthcare background. Many diabetic patients coming from a lower socioeconomic status or those with limited education struggle to understand the health implications of the HbA1c test when they receive this method of explanation, and do not respond to it by improving healthcare compliance. The field of diabetes was already calling for improving health literacy in diabetic patients by developing ‘thinking outside the box’ approaches [34,35], and a recent publication suggests tailoring such messaging based on the psychological phenotyping of patients [36]. This need was also orchestrated in a study which called for the need for long-lasting education for adult diabetic patients toward improving behavior and achieving self-management of diabetes [37]. The ABCDE approach was created as a clinical approach intended to use such an ‘outside the box’ approach by using practical analogies to communicate basic concepts of diabetes in a simplified format which could be immediately understood by everyone, and not be limited by socioeconomic status, linguistic barriers or racial or ethnic backgrounds, or the educational level of diabetic patients, because the analogies were chosen to reflect common, everyday activities in their lives that diabetic patients could readily relate to. For instance, most people are aware that speed limits exist in school zones, and understand that filling diesel in a gasoline car spells trouble, and that it is not possible to fill a bucket with holes in the bottom, but when these simple-to-understand concepts are linked to HbA1c, avoiding sugary beverages and compliance with a healthy diet, there is an epiphanic moment of realization of what these concepts mean for their diabetes. This is perhaps best illustrated by the ‘chair analogy’, where the simple idea that a chair with one broken leg is unstable and can topple is able to communicate basic but powerful concepts of dietary compliance, the value of exercise, and compliance with medication. The ABCDE approach was created, not to replace formal diabetes education, but rather to facilitate an understanding of established components of diabetes education. The ABCDE approach uses the concept of motivational interviewing [38,39,40,41] in an effort to educate, encourage and empower patients with the information and knowledge they need for self-management of diabetes to prevent complications, and live a healthy life. The information communicated by the ABCDE is identical to that provided by certified diabetes educators in formal diabetes classes, but it uses a simpler analogy-based approach to communicate it. A study evaluating the use of communication techniques by diabetes educators found that the use of simple language was frequently reported in diabetes education, and that educators with prolonged experience used more educational techniques than those with less experience [42]—the ABCDE fits well with these findings as it provides a novel, simple and effective means of diabetes education which transcended linguistic barriers among the patients in this retrospective report. This manuscript introduces the ABCDE approach and reports a highly significant improvement in glycemic outcomes over the short-term of 3 months and longer-term of 6 months after receiving the ABCDE. The ABCDE concept holds promise in improving behavior, motivation, health literacy and glycemic outcomes in diabetic patients to help attain glycemic targets, and could be a valuable and inexpensive method of improving adherence with diabetes self-care.

### 4.4. Limitations of ABCDE

The main limitations of this study are: (a) the results are from a retrospective chart analyses; (b) the ABCDE was limited to a HIV-infected, underserved diabetic population; (c) there were no formal assessments of patient understanding of diabetic concepts, patient motivation or behavioral modifications. However, the significant improvement of glycemic parameters with ABCDE without any changes in medication strongly suggests that the ABCDE approach could be a viable approach to improve patient education and glycemic outcomes based on improving understanding about basic concepts of diabetes, at least in underserved populations with socioeconomic barriers, and warrants additional research. 

### 4.5. Future Directions 

We will be to: (a) conduct prospective research studies to test and confirm the validity of ABCDE, and include questionnaires to quantify patient understanding and behavioral modifications; (b) conduct this in underserved and normal populations of diabetic patients; (c) test whether improvements can be sustained over a longer duration of time; (d) continue to develop and include newer analogies of ABCDE to address more concepts in diabetes to make ABCDE even more comprehensive and simple.

## 5. Conclusions

The ABCDE concept was developed to help patients understand diabetes in a relatable manner and thereby improve adherence to all aspects of diabetes management toward reaching glycemic goals. This manuscript provides early proof-of-concept that ABCDE was highly successful in rapidly lowering hyperglycemia, and that the benefits were sustained over a longer duration, suggesting behavior modification. The ABCDE concept is successful in the underserved population of HIV-infected patients with diabetes, but there is need for prospective research to validate and confirm these observations on the ability of ABCDE to improve understanding and modify behavior to improve glycemic outcomes in patients with diabetes.

## Figures and Tables

**Table 1:** **Glycemic indices.** Values are means ± SD; means are statistically different at *p* < 0.05. Visit-1 = first visit where ABCDE was provided; Visit-2 and Visit-3 = visits 3- and 6 months after Visit-1.

	Visit-1	Visit-2 *Visit-1* vs. *Visit-2* *Percent Decrease*	Visit-3 *Visit-1* vs. *Visit-3* *Percent Decrease*
HbA1c %	10.2 ± 1.7	7.3 ± 1.2 *p < 0.0001; 28.7%*	6.8 ± 0.6 *p < 0.0001; 33.1%*
Estimated average glucose (mg/dL)	245.8 ± 47.9	163.3 ± 31.9 *p < 0.0001; 33.6%*	148.1 ± 19.9 *p < 0.0001; 39.8%*
Fasting blood glucose (mg/dL)	264.5 ± 89.8	127.0 ± 38.5 *p < 0.0001; 52.0%*	108.5 ± 17.7 *p < 0.0001; 59.0%*

## Data Availability

All data are contained in this manuscript.
